# Demonstration and Acceptability of a Safer Conception Intervention for Men With HIV in South Africa: Pilot Cohort Study

**DOI:** 10.2196/34262

**Published:** 2022-05-04

**Authors:** Lynn T Matthews, Christina Psaros, Mxolisi Mathenjwa, Nzwakie Mosery, Letitia Rambally Greener, Hazar Khidir, Jacquelyn R Hovey, Madeline C Pratt, Abigail Harrison, Kara Bennett, David R Bangsberg, Jennifer A Smit, Steven A Safren

**Affiliations:** 1 Division of Infectious Diseases Department of Medicine University of Alabama at Birmingham Birmingham, AL United States; 2 Behavioral Medicine Program Department of Psychiatry Massachusetts General Hospital, Harvard Medical School Boston, MA United States; 3 MatCH Research Unit Department of Obstetrics and Gynaecology University of the Witwatersrand Durban South Africa; 4 Population Services International Johannesburg South Africa; 5 Harvard Combined Residency Program in Emergency Medicine Boston, MA United States; 6 School of Medicine University of Alabama at Birmingham Birmingham, AL United States; 7 Department of Behavioral and Social Sciences School of Public Health Brown University Providence, RI United States; 8 Bennett Statistical Consulting Ballston Lake, NY United States; 9 School of Public Health Oregon Health Sciences University - Portland State University Portland, OR United States; 10 Department of Psychology University of Miami Miami, FL United States

**Keywords:** men with HIV, HIV prevention, safer conception, U=U, treatment as prevention, reproductive health, South Africa

## Abstract

**Background:**

Many men with HIV (MWH) want to have children. HIV viral suppression minimizes sexual HIV transmission risks while allowing for conception and optimization of the health of men, their partners, and their infants.

**Objective:**

This study developed and evaluated the feasibility and acceptability of an intervention to promote serostatus disclosure, antiretroviral therapy (ART) uptake and adherence, and viral suppression among MWH who want to have children in South Africa.

**Methods:**

We developed a safer conception intervention (*Sinikithemba Kwabesilisa* or *We give hope to men*) to promote viral suppression via ART uptake and adherence, HIV serostatus disclosure, and other safer conception strategies for MWH in South Africa. Through 3 counseling and 2 booster sessions over 12 weeks, we offered education on safer conception strategies and aided participants in developing a safer conception plan. We recruited MWH (HIV diagnosis known for >1 month), not yet accessing ART or accessing ART for <3 months, in a stable partnership with an HIV-negative or unknown-serostatus woman, and wanting to have a child in the following year. We conducted an open pilot study to evaluate acceptability based on patient participation and exit interviews and feasibility based on recruitment and retention. In-depth exit interviews were conducted with men to explore intervention acceptability. Questionnaires collected at baseline and exit assessed disclosure outcomes; CD4 and HIV-RNA data were used to evaluate preliminary impacts on clinical outcomes of interest.

**Results:**

Among 31 eligible men, 16 (52%) enrolled in the study with a median age of 29 (range 27-44) years and a median time-since-diagnosis of 7 months (range 1 month to 9 years). All identified as Black South African, with 56% (9/16) reporting secondary school completion and 44% (7/16) reporting full-time employment. Approximately 44% (7/16) of participants reported an HIV-negative (vs unknown-serostatus) partner. Approximately 88% (14/16) of men completed the 3 primary counseling sessions. In 11 exit interviews, men reported personal satisfaction with session content and structure while also suggesting that they would refer their peers to the program. They also described the perceived effectiveness of the intervention and self-efficacy to benefit. Although significance testing was not conducted, 81% (13/16) of men were taking ART at the exit, and 100% (13/13) of those on ART were virally suppressed at 12 weeks. Of the 16 men, 12 (75%) reported disclosure to pregnancy partners.

**Conclusions:**

These preliminary data suggest that safer conception care is acceptable to men and has the potential to reduce HIV incidence among women and their children while supporting men’s health. Approximately half of the men who met the screening eligibility criteria were enrolled. Accordingly, refinement to optimize uptake is needed. Providing safer conception care and peer support at the community level may help reach men.

**Trial Registration:**

ClinicalTrials.gov NCT03818984; https://clinicaltrials.gov/ct2/show/NCT03818984

**International Registered Report Identifier (IRRID):**

RR2-https://doi.org/10.1007/s10461-017-1719-4

## Introduction

Worldwide, many men with HIV want to have children [1-5]. For men with HIV who want to father a child with an HIV-negative partner, antiretroviral therapy (ART)–mediated viral suppression effectively eliminates the risk of sexual HIV transmission while improving their health and life span. In South Africa, an estimated 78% of men with HIV know their serostatus, 67% of those who know their status are on ART, and 82% of those men (42% of men with HIV) are virally suppressed [6]. Population cohorts observed large declines in HIV incidence among men in the Test and Treat era in South Africa; however, incidence declines among women lag, largely because of limits in ART uptake and retention of care among men [7,8]. The promise of Test and Treat to reduce individual-level incidence has not yet translated into population-wide benefits [9], and Treatment as Prevention trials only highlight the challenges of reaching, engaging, and retaining men in care [10-13].

Safer conception care is an important strategy for decreasing HIV transmission within the context of intended conception among HIV-serodifferent couples. Counseling and promotion of ART-mediated viral suppression for partners with HIV, pre-exposure prophylaxis (PrEP) for partners without HIV, and education regarding condomless sex timed to peak fertility can dramatically reduce the likelihood of sexual or perinatal transmission of HIV among individuals and couples aiming to conceive [14,15]. However, the reproductive goals of people with HIV, especially men, are not discussed as part of routine clinical care. Men with HIV who want to have children feel stigmatized and are unlikely to ask providers for advice [16,17], and few people with HIV are aware of the opportunities to meet reproductive goals without HIV transmission [18-21].

Worldwide, many decisions around conception and HIV prevention are not equally decided between heterosexual partners, with the determination often made by men [22,23]. Most reproductive health interventions appropriately focus on women; however, providing reproductive health interventions for both men and women is critical to ensuring the health of men with HIV and their families [24]. Therefore, based on formative work with people living with or exposed to HIV in KwaZulu-Natal, South Africa, we developed a male-focused intervention based on cognitive behavioral therapy (CBT) skills to encourage and support men with HIV who want to have children to adopt safer conception behaviors, including HIV serostatus disclosure and initiation of ART (trial registration: ClinicalTrials.gov NCT03818984). The development of the intervention was described previously [25] and was focused on men as the primary intervention recipient while encouraging the inclusion of female partners to optimize their opportunities for testing, treatment, and prevention in the context of their reproductive goals. This study describes an open pilot intervention consistent with Phase Ib of the Obesity-Related Behavioral Intervention Trials (ORBIT) model for behavioral intervention development [26]. We evaluated the feasibility and acceptability of the *Sinikithemba Kwabesilisa* (*We give hope to men*) intervention to offer comprehensive safer conception counseling to men with HIV planning to have a child with an HIV-negative or unknown serostatus partner in South Africa. We also explored the impact of the intervention on ART uptake, adherence to ART, viral suppression, and HIV serostatus disclosure to inform proof of concept for clinical impact and future study planning.

## Methods

### Study Site

Participants were recruited from a collaborative clinic between the AIDS Healthcare Foundation nongovernmental organization and the South Africa Department of Health in Umlazi near Durban, South Africa. Umlazi is a township community 10 miles southwest of the Durban City Center, where HIV prevalence among antenatal clinic attendees is estimated at 42%, and HIV incidence is approximately 6.4 per 100 woman-years [27].

### Inclusion and Exclusion Criteria

Eligible men were male-identifying, aged 20 to 45 years, accessing ART for <3 months (including not on ART), and HIV-positive. In the first phase of recruitment, men were eligible if they had known their serostatus for at least 6 months. Due to recruitment challenges, this criterion was relaxed to knowing their status for at least 1 month. They reported wanting to have a child in the next year with a stable female partner (together for at least 6 months) and that their desired pregnancy partner was HIV-negative or of unknown serostatus and not known to be pregnant. Eligible men had access to a mobile phone and were fluent in either English or isiZulu.

Pregnancy partners of the enrolled men were invited to participate. Eligibility criteria for female partners included age ≥18 years, identifying as a woman, and partnering with an enrolled man (using a couples verification screening tool [28]). In addition, as we did not want the study team to inadvertently disclose a male participant’s serostatus to their pregnancy partner, eligible female partners reported knowing their male partner’s HIV-positive serostatus. Men were recruited independently of their plans or expectations to include their pregnancy partners.

Exclusion criteria for men and women included planning to relocate to a location incompatible with study participation in the next 12 weeks, active drug or alcohol use or active illness requiring treatment within 30 days before study entry that, in the opinion of the research team, could interfere with study participation.

### Recruitment

All men at the clinic site (for HIV care, HIV counseling and testing, sexually transmitted infection [STI] testing, and medication collection) were approached. Men were also invited to participate through mobile vans organized by the same clinic site to test for HIV across the clinic catchment area. Those interested in the study were screened for inclusion, and eligible men were invited to provide informed consent to enroll in the study. Men who claimed interest but did not complete the screening at that time because of other commitments or concerns were provided with a study flyer that included information about the study and contacts for the recruitment team. Interested men could also provide their contact details in a locked study box, one of which was at the clinic at all times and one of which circulated with the mobile testing unit. The study team contacted potential participants for screening and scheduled appointments for enrollment.

### Intervention Content

We conceptualized the design of our intervention based on formative research studies conducted between 2010 and 2015 with men with HIV, women with recent pregnancies with an HIV-serodifferent partner, and providers at public sector clinics in the eThekwini District, South Africa. The process for developing the intervention was described in Khidir et al [25].

**Figure 1:**
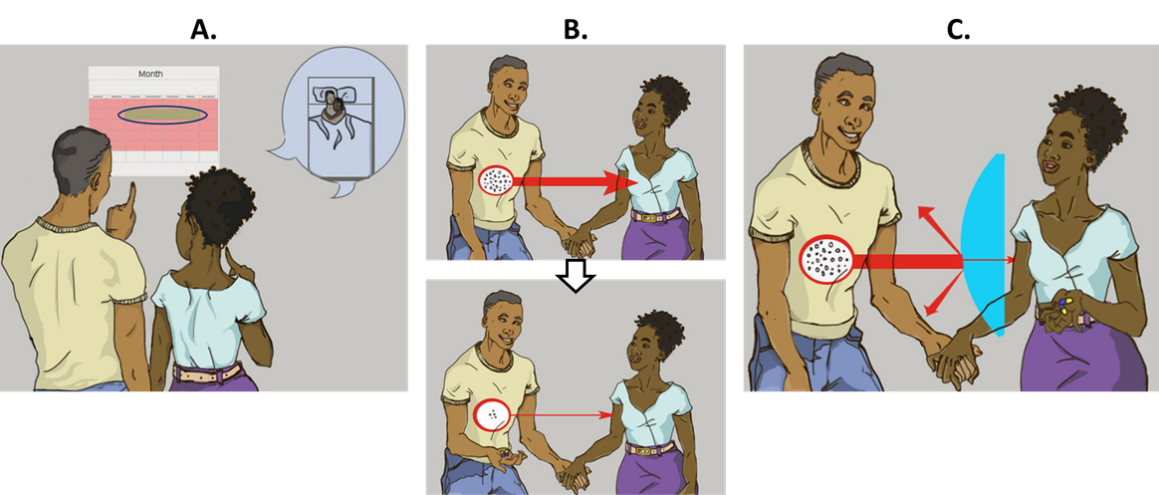
Locally relevant images used to present key safer conception strategies such as (A) timed condomless sex to peak fertility, (B) Treatment as Prevention, and (C) pre-exposure prophylaxis for the HIV-negative partner.

As described previously, although men maintain a great degree of relationship power related to decisions about conception, they are less involved in reproductive health care counseling, which results in poor outcomes for both men and women. Thus, our study recruited men as individuals in an attempt to close this gap. We aimed to promote safer conception strategies, development of a safer conception plan, planning and problem solving around plan execution, and communication and HIV serostatus disclosure training for a broad population of men with HIV, including those who might not yet be in a mutually disclosed partnership. On the basis of our findings that men with HIV have limited knowledge of HIV serodifference and safer conception strategies, the intervention provided comprehensive safer conception education based on safer conception strategies recommended by international and national guidelines, including (1) Couples HIV Counseling and Testing (CHCT) and other strategies for serostatus disclosure, (2) initiation of ART and delaying condomless sex until achieving viral load suppression (Treatment as Prevention), (3) timing condomless sex to peak fertility, (4) treatment of STIs, (5) sperm washing and in vitro fertilization, and (6) adoption. HIV PrEP was not yet available in or recommended as part of safer conception care in South Africa at the time this study was conducted. We developed a counseling tool that integrates relevant illustrations to simplify and explain key medical concepts (Figure 1) [16].

**Figure 2:**
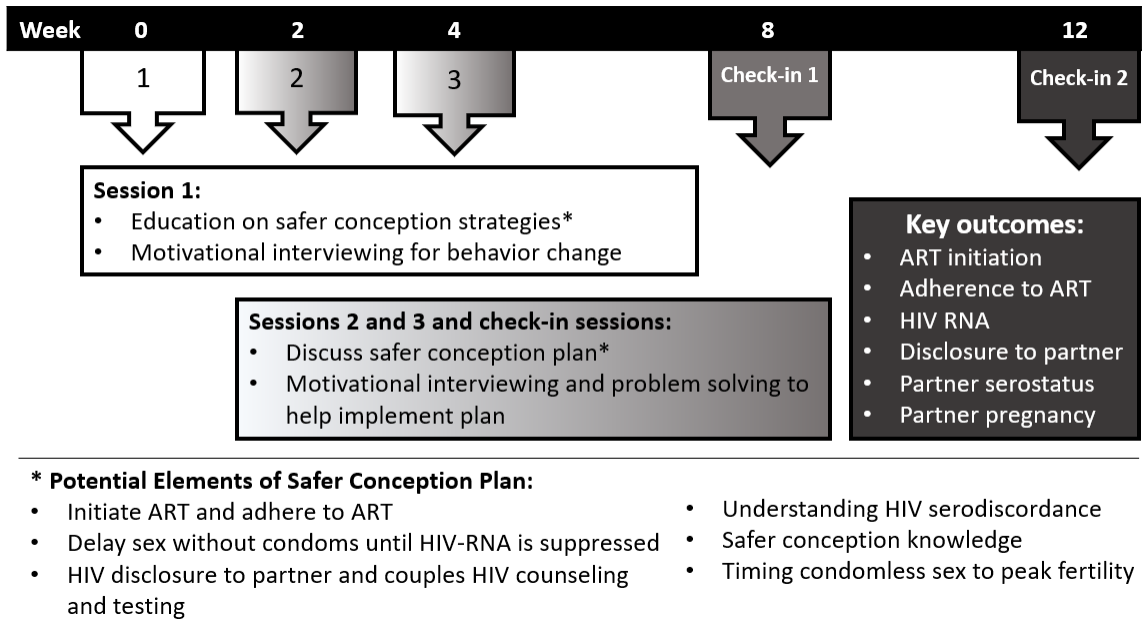
Diagram highlighting Sinikithemba Kwabesilisa session content over 12 weeks and key outcomes (adapted from Mathenjwa et al [16], which is published under Creative Commons Attribution 4.0 International License [37]). ART: antiretroviral therapy.

The behavioral component of the intervention (led by CP and SAS) used strategies drawn from CBT and included a brief motivational exercise, problem solving, and communication skills training [29-31]. The *Life-Steps* intervention was also included to support medication adherence [30]. Communication skills training was based on elements from the Stepping Stones [32-34] and Horizons programs developed in South Africa and aimed to foster forthright and respectful communication between partners and support participants in negotiating disclosure and reproductive goals [35,36]. The multisession intervention comprised 3 core sessions and 2 follow-up sessions to allow men time to update their safer conception plans while accessing support from the study as changes were implemented. The 3 core sessions at weeks 0, 2, and 4 offered education on safer conception strategies and motivational interviewing for behavioral change. Follow-up sessions at 2 and 4 weeks and check-ins at 8 and 12 weeks included a review of safer conception plans and motivational interviewing and problem solving to execute the plan. This also allowed time for men to disclose their serostatus, think through prevention options, and learn skills to communicate with their partners regarding reproductive goals. Session content is outlined in Figure 2.

### Intervention Delivery

#### Mechanism of Delivery

The manualized CBT intervention was facilitated by 1 of 3 South African men with fluency in both isiZulu and English, training in HIV counseling, and training in the intervention strategies (Figure 2). A female interventionist with >5 years’ experience as an HIV, sexual, and reproductive health researcher with training in HIV counseling and testing was also trained to conduct sessions that included female partners. Interventionists were trained in couples-based HIV counseling and testing by the Centers for Disease Control and Prevention in South Africa [38]. Intervention content training was provided through several in-person sessions: content related to safer conception behaviors was led by LTM, and CBT-based content was led by CP. Training modules included basic counseling skills (nonjudgmentalism, active listening, and reflection), evidence for the use of CBT to support behavior change, and training in skills specific to the intervention (eg, motivational interviewing strategies, problem solving, and communication skills). The training included both didactic components and experiential exercises. Education on gender norms and safer conception strategies was also included.

#### Intervention Fidelity

Interventionists completed standardized training on the intervention manual, filled in session checklists detailing the completion of key intervention components for each session, and completed a same-day debriefing session with a team member trained in the intervention and with a master’s degree in research psychology to review session goals and content. Sessions were audio recorded, with a subset of sessions translated into English and transcribed. The transcripts and interventionist notes were reviewed independently by CP during supervision sessions, and feedback was provided.

#### Interventionist Supervision

Supervision, conducted biweekly via web conferencing, was provided by the study psychologists and intervention developers. Supervision involved a review of cases, discussion, and problem solving of clinical challenges, planning of subsequent sessions, and general support for the interventionists.

### Data Collection

#### Questionnaire

A face-to-face questionnaire was completed at enrollment and exit with enrolled men and women by a researcher distinct from the interventionist. Demographic data, HIV clinical data (diagnosis date and clinical history), and reproductive health history (number of prior partner pregnancies, number of prior live births, number of living children, and HIV status of children) were collected.

Questionnaires also assessed disclosure to partners and other contacts using a sexual networks instrument to assess the full spectrum of disclosure [39,40]. Personal and partner fertility desires were assessed using the Centers for Disease Control and Prevention Pregnancy Risk Assessment Monitoring System instrument [41-44]. We asked participants about safer conception plans, whether they had completed couples-based HIV counseling and testing, and partner pregnancy.

On the basis of our conceptual framework [45], we measured the potential mediators and moderators of the outcomes. HIV knowledge was assessed using the brief HIV Knowledge Questionnaire [46]. Stigma toward HIV was measured using the Internalized AIDS-Related Stigma Scale [47]. We measured attitudes toward gender relations using the Gender Equitable Men scale, validated in KwaZulu-Natal [48-51]. This scale includes 24 items in 2 subsets. Subset 1 measures inequitable gender norms and includes items such as “It is the man who decides what type of sex to have.” Subset 2 measures equitable gender norms and includes items such as “A couple should decide together if they want to have children.” Response options are *agree*, *partially agree*, and *disagree*. When combined, the scores can be used as a continuous variable or categorized as low equity=1 to 23, moderate equity=24 to 47, and high equity=48 to 72.

We used the Decision-Making Dominance (DMD) subscale of the Sexual Relationship Power Scale. This 8-item subscale comprises questions such as, “Who usually has more to say about whether you have sex?” The DMD subscale was constructed to measure the balance of decision-making power (1=your partner has more power, 2=both of you have equal power, and 3=you have more power) on each of the 8 items, with higher scores indicating higher relationship power for the respondent. DMD scores were totaled and divided by the number of nonmissing items to calculate the mean individual score. The responses subsequently indicate whether the participant, their partner, or both equally demonstrate DMD. We also described the number of participants with *high* (>2.82), *medium* (2.82-2.43), and *low* (<2.43) levels of DMD [28,52].

We assessed social support using a 10-item subscale of the Social Support Inventory [53,54]. Alcohol consumption was assessed using the 10-item Alcohol Use Disorders Identification Test questionnaire, with questions labeled 0 to 4 and a maximum score of 40 [55]. Drug use was assessed using the Alcohol and Substance Involvement Screening Test interview questionnaire [56]. We screened for depression and anxiety using the Hopkins Symptom Checklist [57].

#### ART Adherence

For men taking ART, adherence to therapy was measured using the Medication Event Monitoring System (MEMS; Aardex), a bottle cap with a chip that clocks openings, downloaded at study visits [58] after ART initiation. Adherence was defined as the proportion of days with at least one bottle opening during the follow-up period.

#### Sexual Behavior

Sexual behavior was assessed through weekly password-protected SMS text messaging to request participant reports on intercourse frequency and timing, condom use, and partners. These data were collected to calculate secondary outcomes, including the proportion of men who delayed sex without condoms until viral load suppression, or limited sex without condoms to peak fertility.

#### Medical Record Review

Research assistants collected data on CD4 cell count at enrollment (viral load would not yet have been collected for men meeting inclusion criteria) and HIV viral load at 12 weeks after commencing ART from the medical record. Viral load assessment as early as 8 weeks after initiating therapy was not part of the standard of care but organized for this pilot intervention, given the 12-week follow-up period.

#### Exit In-depth Interviews

Exit interviews were conducted with 11 men and 1 female partner to explore perceptions of intervention delivery logistics, intervention content, female partner participation, HIV serostatus disclosure, and a participant-centered evaluation of their participation. The interviews lasted for approximately 60 to 90 minutes. The interviews were audio recorded, transcribed when indicated, and translated into English.

### Outcomes and Analysis

#### Measurement of Outcomes of Interest

We used the ORBIT model for the development of behavioral interventions to frame this phase of the research as Phase Ib preliminary testing to refine the intervention [26]. Primary outcomes of interest include those described in the following sections.

##### Feasibility

The proportion of men screened who were eligible to participate and the proportion eligible who elected to participate are described. We also describe the proportion of men who completed the 3 primary intervention sessions.

##### Acceptability

Acceptability was inferred based on client participation. In addition, in-depth interviews were conducted with a subset of men to explore participant experiences with the intervention. We report on codes categorizing client perceptions of acceptability based on their experience with the intervention, including affective attitude (codes included *satisfaction with session content* and *satisfaction with number of sessions*), perceived efficacy (*benefits of the safer conception program* and *peer referral to the program*), and self-efficacy to benefit (*what makes participation easier* and *community demand*) [59]*.*

Owing to the small sample size and open pilot design, we were not powered to evaluate HIV-related outcomes. However, in line with the ORBIT framework and to inform future trial planning, we describe the clinical outcomes of interest discussed in the following sections.

##### Disclosure

We measured disclosure based on participant responses to an item in our questionnaire: “Does your partner know your HIV-status?” As disclosure is a complex process that is subject to desirability bias, and some partnerships changed, we did not censor men who reported disclosure at enrollment from reporting disclosure at the exit interview.

##### HIV-RNA Viral Suppression

As the pilot intervention was brief, we provided HIV-RNA testing to men 8 weeks after initiating therapy, which was not the standard of care. Therefore, our outcome for viral suppression was a combined outcome of the proportion of men retained in the study who suppressed viral load. We also describe the proportion of all men who initiated therapy for at least 8 weeks who achieved viral suppression (including all men in the denominator even if they did not remain in the study long enough to have a viral load assessed).

##### ART Uptake

Men who were prescribed ART in their medical records were categorized as initiating ART. The start of ART was corroborated by the men in the intervention sessions.

##### ART Adherence

MEMS caps were used to measure daily pill-taking behavior for all men who initiated ART with medication bottles that fit the MEMS cap. As we did not provide ART, some men received ART in nonstandard pill bottles that did not accommodate the MEMS cap size.

#### Quantitative Analysis

The baseline characteristics of the participants and outcomes were described. SAS analytic software (SAS Institute Inc) was used to conduct all quantitative analyses.

#### Qualitative Analysis

Transcripts were reviewed by several members of the research team (MM, LTM, HK, and LG). Using an inductive and deductive approach informed by an ecological conceptual framework [45], we developed a codebook to organize text into categories. Data were organized using thematic analysis [6]. The thematic analysis results have been described elsewhere [16]. This manuscript reports findings from categories regarding intervention acceptability (positive and negative codes), including affective attitudes toward the intervention, perceived efficacy, and self-efficacy to benefit from the intervention. [59].

### Ethics Approval

The competing concerns regarding respecting the privacy of male participants and doing our best to protect female partners were carefully considered [60]. We created letters for men to give their pregnancy partners, informing them of the risk of acquiring HIV and encouraging them to seek HIV counseling and testing. Men were reminded that these letters would effectively disclose their HIV serostatus to their partners. We encouraged men to include their female partners in counseling sessions to encourage the transfer of safer conception counseling to partners, although we recognized that not all men, particularly those unable to disclose, would be willing to do this. We created a separate information session led by a female research assistant for the female partners who chose to participate. Ethics approval for the study was obtained from the human research ethics committee at the University of Witwatersrand (Johannesburg, South Africa; approval#M150426) and the institutional review board at Partners Healthcare (Boston, United States; approval#2013P002693). Site permission was also obtained from the study clinic.

## Results

### Enrollment

**Figure 3:**
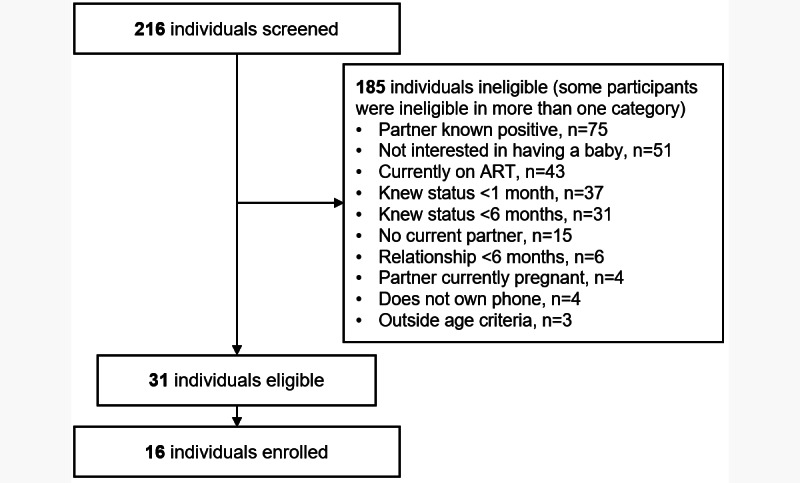
CONSORT (Consolidated Standards of Reporting Trials) diagram for screening and enrollment of men. ART: antiretroviral therapy.

Between November 2015 and December 2017, 216 men were screened. Of the 31 eligible men, 16 (52%) were enrolled in this study. The remaining 48% (15/31) of eligible men were not enrolled as they did not answer or return phone calls, did not attend scheduled appointments, or articulated that personal business and/or life circumstances made it difficult to schedule an appointment. The most common reasons for ineligibility were reporting a pregnancy partner living with HIV (75/216, 34.7%) or not wanting to have a child in the next year (51/216, 23.6%). In addition, 19.9% (43/216) of men were excluded based on ART use (initially, any ART use was an exclusion criterion; this was later modified to include those with ART use for <3 months). Approximately 14.4% (31/216) of men were excluded based on knowing their HIV serostatus for <1 month and 31/216 (14.3%) were excluded based on knowing their serostatus for less than six months (the original serostatus knowledge criterion). Approximately 9.7% (21/216) of men were excluded based on not being in a stable relationship for at least 6 months. Additional exclusions are noted in Figure 3.

Among the 16 enrolled men, the median age was 29 years (IQR 24-44); all were Black South African, 56% (9/16) had completed high school or above, and 44% (7/16) had full-time employment. Men had been living with HIV for a median of 1.7 years. At the time this work was completed, the standard of care included checking the viral load at 6 months after treatment initiation but not at baseline [61]. Therefore, all enrolled men who had access to ART for 0 to 3 months had no viral load data and were unlikely to be virally suppressed because of this timing. Enrolled men reported a median of 1.5 sexual partners in the prior 3 months. Most participants (13/16, 81%) had children. The desired pregnancy partner was described as a long-term girlfriend by all participants, and no participant reported consistent condom use with this partner. Approximately 44% (7/16) of men reported having disclosed their HIV serostatus to their pregnancy partners at enrollment (Table 1).

A total of 3 women partnered with the index male participants enrolled with a median age of 27 years, all of whom were Black South African women reporting 1 sexual partner. All women who approached the research team were eligible to enroll; women were enrolled 1 to 3 months after the male index was enrolled (Table 1).

Half of the enrolled men (8/16, 50%) and all of the women scored within the top one-third for gender-equitable responses. In addition, most of the enrolled men (15/16, 94%) reported equal decision-making power between themselves and their partners. Only one of the men (1/16, 6%) had discussed having children with a health care worker or counselor since knowing their HIV serostatus. Of note, a large proportion of participants screened positive for depression, including 31% (5/16) of men and 67% (2/3) of women (Table 2).

**Table 1:** Enrollment characteristics for men (N=16) and women (N=3) pregnancy partners.

Variable			Men	Women
Age (years), median (range)			29.7 (24.2-44.6)	27.0 (25.5-31.7)
Race (Black South African), n (%)			16 (100)	3 (100)
HIV serostatus positive, n (%)			16 (100)	2 (67)^a^
Years living with HIV, median (range)			1.7 (0.1-8.8)	0.1 (0-0.2)^b^
**Education completed, n (%)**				
	Some secondary		7 (44)	1 (33)
	Completed secondary or above		9 (56)	2 (67)
**Employment, n (%)**				
	Not employed		6 (38)	2 (67)
	Part-time employed		2 (13)	0 (0)
	Self-employed		1 (6)	0 (0)
	Full-time employed		7 (44)	1 (33)
Number of sexual partners in the past 3 months, median (range)			1.5 (1-3)	1 (1-1)
**How many children fathered/born? n (%)**				
	0		3 (19)	0 (0)
	1-3		10 (62)	3 (100)
	≥4		3 (19)	0 (0)
**Pregnancy partner characteristics as reported by index male participant**				
	Age (years)^c^, median (range)		25 (21-37)	—^d^
	Male age—pregnant partner age, median (range)		3.0 (−0.7-9.6)	—
	Children with this partner^b^, median (range)		1 (1-1)	—
	Partnership type (long term), n (%)		16 (100)	—
	Condomless sex at last report, n (%)		4 (25)	—
	Consistent condom use, n (%)		10 (63)	—
	Anal sex, n (%)		0 (0)	—
	**HIV status, n (%)**			
		Do not know	6 (38)	—
		Negative	9 (56)	—
		Positive	1 (6)	—
	Disclosed to her, n (%)		7 (44)	—
	**She wants to have a baby with you, n (%)**			
		Yes	15 (94)	—
		Do not know	1 (6)	—
Alcohol use (maximum score possible=40), median (IQR)			3.5 (2-9.5)^e^	0 (0-0)^f^
Drug use (maximum score possible=52), median (IQR)			4 (0-7)^g^	0 (0-0)

^a^A total of 2 female partners were living with HIV at the time of enrollment: 1 was diagnosed before enrollment, and 1 was diagnosed at enrollment.

^b^n=2.

^c^n=15.

^d^Characteristics reported by male participants only.

^e^Minimum=0 and maximum=18.

^f^n=1.

^g^Minimum=0 and maximum=9.

**Table 2:** Additional baseline characteristics that factor into our safer conception behavior conceptual framework.

Variable		Men (N=16)	Women (N=3)
Major depression by HSCL^a^, n (%)		5 (31)	2 (67)
HIV knowledge, median (range)		10.5 (6-13)	1 (0-2)
**DMD^b^ of SRPS^c,d^**			
	Values, median (IQR)	2.25 (2.00-2.56)	1.75 (1.62-2.00)
	Proportion high (>2.82), n (%)	1 (6)	0 (0)
**Gender-equitable men^e^**			
	Values, median (IQR)	36.5 (30.00-42.77)	33 (32-40)
	Proportion high (25-36 points), n (%)	8 (50)	3 (100)
Have you ever had a conversation with a health care worker or counselor about having children (since knowing your status)? n (%)		1 (6)	0 (0)
Safer pregnancy knowledge score (maximum score possible=10), median (IQR)		6 (4-7)	4 (4-5)
AIDS-related stigma (maximum score possible=6)^f^, median (IQR)		3 (2-4)	N/A^g^
Social support (maximum score possible=4), median (IQR),		3.75 (3.45-4.00)	N/A

^a^HSCL: Hopkins Symptom Checklist.

^b^DMD: decision-making dominance.

^c^SRPS: Sexual Relationship Power Scale.

^d^Items on the DMD Factor subscale are scored as 1=your partner, 2=both of you equally, and 3=you.

^e^A total of 3 points for each response representing gender equality, 2 points for each response representing moderate gender equality, and 1 point for each response representing the lowest equality. The average score on the Gender Equitable Men scale is calculated by summing the points scored by each respondent and dividing by the total number of respondents.

^f^n=15; missing responses for n=1.

^g^N/A: not applicable.

### Fidelity to Intervention Delivery

On the basis of a review of recorded sessions and session checklists, there was high fidelity to the intervention delivery, with interventionists routinely adhering to the manual and covering all the modules in each recorded session.

### Outcomes

#### Feasibility

As stated previously, 7.4% (16/216) of the screened men were enrolled in the study. The main reasons for not meeting the eligibility criteria were generally related to the intervention not being applicable to their current situation: partners already knowing their status, not interested in having a baby, already on ART, knowing HIV status for shorter or longer than the study window, or not having a current partner. The main reason for eligible nonenrollments was related to challenges in scheduling.

Intervention sessions varied in length, although among the recorded sessions, all sessions were at least 40 minutes in length. The longest session was 75 minutes in length.

For the 3 women who enrolled, each participant completed 1 baseline intervention session.

#### ART Use

Of the 16 men, 13 (81%) were receiving ART upon exiting the study. Approximately 75% (9/12 with nonmissing data at week 12) of men reported disclosure of serostatus to their partner, including 56% (5/9) who reported participating in couples-based HIV counseling and testing. Among men accessing ART, contributing a median of 35 (IQR 28-85) days of MEMS cap follow-up with adherence, men took a median of 89% (range 67%-100%) of doses. Of the 8 men with adherence data, 6 (75%) took at least 80% of doses.

#### Viral Suppression

Among those for whom HIV-RNA was sampled, all (7/7, 100%) achieved viral suppression by 12 weeks. Approximately 13% (2/16) of men who started ART during the sessions were lost to study follow-up. An additional 25% (4/16) of men were not yet eligible to have their HIV-RNA checked, given <8 weeks since ART initiation at the end of the study.

#### Sexual Behavior

Approximately 44% (7/16) of men participated in the SMS text messaging surveys, responding to a median of 93% (range 63%-100%) of messages. Of these 7 men, none attempted to time condomless sex to peak fertility based on their selections. Table 3 shows the other safer conception methods that men selected.

**Table 3:** Safer conception method selection and outcomes by participant.

Participant ID	Number of sessions completed^a^	Strategies endorsed	Disclosure at 12 weeks	ART^b^ uptake	Adherence >80%	Median Adherence % (days of adherence data)	Viral suppression at 12 weeks
M1001	5	ART initiation Timed sex	No	Yes	Yes	97 (30 days)	Yes
M1002	5	ART initiation Timed sex Pick one pregnancy partner	No	Yes	N/A^c^ (initiated ART at last session)	N/A (initiated ART at last session)	N/A (initiated ART at last session)
M1003	5	Timed sex Disclosure	Yes	Yes	No	96 (24 days)	Yes
M1004	1	Condom use ART Disclosure	LTF^d^	LTF	LTF	LTF	LTF
M1005	5	ART initiation Disclosure	Yes	Yes	Yes	100 (24 days)	Yes
M1006	3	Timed sex Disclosure	Yes	N/A	N/A	N/A	N/A
M1007	5	ART Timed sex Condoms Disclosure	Yes	Yes	N/A (initiated ART at last session)	N/A (initiated ART at last session)	N/A (initiated ART at last session)
M1008	5	Timed sex ART Disclosure	Yes	Yes	Yes	79 (29 days)	VL^e^ not checked as not on ART for <8 weeks
M1009	5	ART Timed sex Disclosure	Yes	Yes	Yes	67 (108 days)	VL not checked as not on ART for <8 weeks
M1010	5	Disclosure ART Timed sex	Yes	Yes	N/A; not on FDC^f^, self-reported high adherence	N/A; not on FDC^f^, self-reported high adherence	Yes
M1011	1	Timed sex Treatment as prevention	N/A	N/A	N/A	N/A	N/A
M1012	5	Timed sex Treatment as prevention Sperm washing	Yes	Yes	Yes	87 (39 days)	Yes
M1013	5	Timed sex Treatment as prevention	Yes	Yes	Yes	91 (85 days)	Yes
M1014	5	Timed sex Treatment as prevention	Yes	Yes	Yes	83 (84 days)	Yes
M1015	5	Timed sex PMTCTg Treatment as prevention	Yes	Yes	Not initiated on MEMS^h^	N/A	Not available
M1016	2	Timed sex PMTCT Treatment as prevention	Yes	Yes	LTF	LTF	LTF

^a^Out of 3 main sessions and 2 booster sessions.

^b^ART: antiretroviral therapy.

^c^N/A: not applicable.

^d^LTF: lost to follow-up.

^e^VL: viral load.

^f^FDC: fixed-dose combination—only the FDC tablets bottle fit the electronic pill cap.

^g^PMTCT: prevention of mother-to-child transmission.

^h^MEMS: Medication Event Monitoring System, electronic pill cap.

#### Acceptability

Of the 16 men, 14 (88%) completed all 3 intervention sessions. We conducted in-depth interviews with 11 enrolled men and 1 female partner at the study exit. Participants described personal satisfaction with session content and structure while also suggesting that they would refer peers to the program and that with time, it became easier to participate in the sessions as they gained trust with the counselor (Textbox 1). They also described the perceived effectiveness of the intervention and self-efficacy to benefit by reporting that the sessions provided new knowledge, instilled hope, and were perceived as helpful overall (Textbox 1).

**Quotes from exit interviews related to the acceptability of the intervention.**

**Personal satisfaction**

- “Everyday if I had come to you guys and discuss, there was always something that I would have gained” [M1002, man with HIV aged 31 years]
- “My brother, I think what is more important for the crisis that we are facing is us as males, we are afraid of coming out onto the open about things, we end up not talking about things. So getting such a programme, perhaps you would start picking up on the third or the fourth sessions…and that is when you start opening up and realising that these are good people and so forth, because we are the kind of people who do not want to be sympathised for...” [M1007, man with HIV aged 35 years]
- “I found it very helpful, because it shows, even though things have been happening in a way that...to be honest I was not comfortable about the whole situation that we were in, but then after we were counselled and told that there are right ways of doing things I thereafter felt great actually...I even began to trust him, my partner, again.” [F1007, woman partnered with M1007]
- “...there are also people outside that I have even been able to explain to them like ‘guys there are things like this in life, if perhaps you are able to have time and go there if you have an HIV-negative woman and you are HIV-positive and have time with those guys and sit with them, there is some information that they will give you so that you can also be proud and realise that you are still a person.’” [M1008 man with HIV aged 34 years]

**Perceived effectiveness of intervention**

- “It has encouraged me that, what is right is that you check and know your status so that you can be able to start treatment if you have the virus and if you are negative you will be able to look after yourself and avoid having to get to a critical stage. I would say the programme has really helped me.” [M1007, man with HIV aged 35 years]
- “And your dream becomes that...and you no longer focus on the idea that you are sick and that life is now on a standstill. When one is here with you guys, hopes come back and you feel hopes revived that you could actually still have babies, you can still be able to plan for your family and so forth. So I would have liked for [the program] to move forward because it will help a lot of people.” [M1013, man with HIV aged 30 years]
- “I think it [the program] had a good impact. It helped me a lot. Psychologically, I was not able to even live, and I feel like a person once again. Because what came through my mind was that I will not by any chance have a baby, so it means I will just die in pain. All those thoughts have been washed away. If you can just take treatment as prescribed.” [M1002, man with HIV aged 31 years]

## Discussion

### Principal Findings

Our findings highlight the acceptability of a clinic-based intervention offering male-centered care to address reproductive goals to engage and retain men in HIV care in South Africa. We describe the first pilot safer conception intervention for South African men with HIV who are not yet virally suppressed and are planning to have a child with a partner of unknown or HIV-negative serostatus. Of the 16 men, 14 (88%) completed all 3 intervention sessions and reported satisfaction with the content. There were promising signals that the intervention supported men to disclose, use ART, and suppress HIV; based on 12-week exit data, 75% (12/16) of men with complete data disclosed HIV serostatus to a partner, 81% (13/16) of men initiated ART, and 100% of those who accessed ART had viral suppression. This is an improvement over the standard of care in South Africa, where only an estimated 78% of men with HIV know their serostatus, 67% of those who know their status are on ART (52% of men with HIV), and 82% of those men (42% of men with HIV) are virally suppressed [6]. The *Sinikithemba Kwabesilisa* open pilot study provides proof of concept that male-centered care that addresses reproductive goals is a promising strategy for engaging and retaining men in HIV care in South Africa, with opportunities to promote men’s health while reducing HIV incidence and perinatal transmission among women of reproductive age.

Gaps in meeting HIV treatment goals for men are well-articulated; to the best of our knowledge, this is the first program to attempt to bridge these gaps by addressing the reproductive goals of men [62,63]. Although some community programs for men have been successful [64-66], we are not aware of other clinic-based programs that provide male-centered HIV care in South Africa. In Lesotho, a male-centered HIV clinic program staffed by male providers has demonstrated high demand and reach [67]. Safer conception care, or programs that aim to reduce HIV transmission in the context of heterosexual HIV-affected couples pursuing reproductive goals, has focused on couples or individual women and has not been aimed at meeting the needs of men [14,68,69]. The empiric question of whether HIV treatment in the context of reproductive goals would be acceptable and feasible for men in South Africa was addressed by this program, in which most men completed the sessions, and exit feedback was positive. This work has also been adapted to a rural Ugandan setting, with encouraging data regarding service uptake and clinical outcomes for men [70].

Men were pleased with the content and structure of the intervention; however, some considerations for further refinement of the intervention emerged. The first was to streamline the content. The intervention included 3 primary sessions and 2 boosters informed by effective behavioral interventions [71]. Most (14/16, 88%) of the men attended all the sessions, and in exit interviews, they articulated wanting more sessions, given the novelty of the information, value of role-plays, and time needed to develop rapport and trust [16]. Given the lack of male-centered care in the health system [72,73], men may have appreciated individual counseling. The interventionists and participants felt that their time together was relatively short, highlighting the importance of focusing on achievable, easy-to-understand prevention strategies that also align with broader HIV treatment and prevention goals [68]. We believe that future iterations of this work can simplify messages to the following essential components: ART for partners with HIV, disclosure, PrEP for partners without HIV, and STI testing and treatment. This would remove the discussion of timing condomless sex to peak fertility as it is challenging and time consuming to teach and implement [74]. Although most men in our cohort included timing condomless sex to peak fertility, none were able to execute this based on sexual behavior SMS text messaging data. A recent study in rural Uganda showed low uptake and poor implementation of this complex strategy [69], and data from systematic reviews suggest that it may not help couples identify peak fertility periods [69,75]. Indeed, given infrequent HIV-RNA monitoring in most resource-limited settings, it may be more practical and better aligned with HIV prevention goals, to time condomless sex to periods of known virologic suppression instead of peak fertility. Another component to consider for removal to allow for streamlining is sperm washing. Multiple studies with diverse populations across sub-Saharan Africa have observed that most people affected by HIV are not motivated to pursue sperm washing and assisted reproductive technologies unless necessary to address infertility [17,76,77].

The intervention was delivered by male lay counselors trained in CBT strategies by the study team. The idea that these concepts could be delivered by counselors, who may have more time than providers, has been proposed [68] and is an important aspect of task shifting to allow stretched health care systems to provide patient-centered care [3]. Counseling by team members who have time to spend with clients is critical—in exit interviews, men reported the need to have time to develop rapport and learn the basics of HIV treatment and prevention, as well as more complex skills such as communication.

Among the enrolled men, most started ART, and adherence was excellent. Of the 16 enrolled men, 13 (81%) were taking ART upon exiting the study, and men took a median of 89% (range 67%-100%) of doses. Among those for whom HIV-RNA was sampled, all (7/7, 100%) achieved viral suppression by 12 weeks. Longer-term adherence and retention in care are challenging for men in South Africa; thus, future iterations of this intervention will include longer-term evaluation of outcomes to measure effectiveness. We believe that these data serve as compelling proof of concept that this may be an important novel strategy for promoting the HIV care cascade for a traditionally difficult to retain and suppress population.

The intervention was successful at promoting disclosure, and the findings provide insights into novel strategies for promoting disclosure among men who have sex with women. At 12 weeks, 75% (12/16) of the men with complete data reported disclosure to their partners. This is based on self-report and is subject to social desirability bias; however, because of the small sample size and the rapport developed with the interventionists, we know that participant disclosure stories shared in the sessions aligned with the survey data. CHCT is a key component of HIV serostatus disclosure interventions. Many of these interventions include group-based sessions or standard HIV counseling and testing, followed by couples counseling sessions with trained health workers [78-80]. A study in KwaZulu-Natal found that only 42% of couples who participated in a couples counseling intervention participated in CHCT within a 9-month follow-up period [70]. Multiple studies have found that factors influencing the uptake of CHCT include the perceived benefits of HIV testing, perception and knowledge of CHCT, suspicions of infidelity, fear of HIV test results, and gender power imbalances [81-83]. Our intervention provided participants with education on CHCT, disclosure counseling, and problem-solving skills. We also supported and role-played individual-level disclosure to partners. Only 3 women attended the clinic for sessions, and they and their partners reported the challenges of accessing CHCT in busy public sector clinics with limited hours for people with full lives. As CHCT does not work well for everyone, we maintain that empowering men to disclose to partners through role-play and supported communication may be an important additional strategy to promote.

The largest challenge to feasibility was recruitment. We screened very few men over the period because of the absence of men in the clinic setting where our intervention was based; only 78% of men with HIV in South Africa know their status, fewer are in care, and low testing is a trend across the continent [84]. The challenges of clinic recruitment highlight the importance of community engagement to reach men who are often not available during clinic hours and who experience or anticipate health care worker stigma at clinics [66,85]. In exit interviews, men discussed that this program was unique in supporting their reproductive goals; ongoing provider stigma toward men with HIV who want to have children may have made other men reluctant to engage in the clinic-based program [16]. Future iterations of this work will require community recruitment to meet men where they are. In exit interviews, men suggested that future work should include broader public health messages; radio spots; and venue-based recruitment at community centers, football clubs, bars, and factories [16]. The intervention may be maximally effective when combined with other efforts to minimize structural barriers to care engagement and retention [66,86-92], reduce health care provider stigma [93], and link HIV care to other needed services [94,95]. We hope to evaluate this approach with a larger sample of men recruited from the community than from the standard of care in future work.

Including a broader range of men in this study could also enhance reach. In this trial, many interested men were ineligible. Approximately 40% (75/185) of the men we excluded reported that their desired pregnancy partner was living with HIV. On the basis of the understandings of disclosure and assumptions that people with HIV make about partner seroconcordance, many men reporting a partner living with HIV may not know their partner’s serostatus but assume concordant serostatus across the partnership [18,80]. Furthermore, seroconcordant-positive couples with HIV and planning for a child can benefit from interventions to promote ART use, viral suppression, STI testing, and treatment for both partners. Future iterations of this work will include men with HIV regardless of partner status and emphasize a serostatus-neutral approach such that enrolled partners can be tested for HIV and linked to HIV care and treatment or prevention (eg, PrEP). Men were also ineligible based on not knowing their serostatus for at least 1 month and not having a stable partnership. Given the near ubiquity of fertility goals and fluidity in partnerships where pregnancies can occur, future iterations of this study will not require these elements.

### Limitations

There are limitations to this pilot study because of the small sample size, men who overcame barriers to enroll and attend sessions, lack of comparison arm, or ability to report on longer-term outcomes. In addition, the qualitative component had a small sample size, and saturation was not met (particularly with respect to input from female partners). Nonetheless, the trial was designed to be an important proof of concept, following ORBIT guidelines as part of intervention development.

### Conclusions

These preliminary data suggest that HIV care that is male-centered and addresses reproductive goals is acceptable to men and has the potential to reduce HIV incidence among women and their children while supporting men’s health. The next steps include adapting the intervention to reach men who have not yet been tested or are not yet in care to evaluate the impact. Future work should include men regardless of partner serostatus; larger-scale randomized projects are needed to evaluate the impact and examine cost-effectiveness. Finally, understanding whether and how health care workers, public health administrators, and other key stakeholders would adopt the elements of this intervention needs to be explored.

## Abbreviations

ART: antiretroviral therapy
CBT: cognitive behavioral therapy
CHCT: Couples HIV Counseling and Testing
DMD: Decision-Making Dominance
MEMS: Medication Event Monitoring System
ORBIT: Obesity-Related Behavioral Intervention Trials
PrEP: pre-exposure prophylaxis
STI: sexually transmitted infection
